# How much does it cost to retain antiretroviral therapy (ART) clients in their first year? Routine financial costs of retention interventions at Lighthouse Trustin Lilongwe, Malawi

**DOI:** 10.21203/rs.3.rs-4939155/v1

**Published:** 2024-10-15

**Authors:** Hiwot Weldemariam, Agness Thawani, Christine Kiruthu-Kamamia, Jacqueline Huwa, Mirriam Chipanda, Hannock Tweya, Caryl Feldacker

**Affiliations:** University of Washington; Lighthouse Trust; Lighthouse Trust; Lighthouse Trust; Lighthouse Trust; University of Washington; International Training and Education Center for Health

**Keywords:** cost of routine health interventions, activity-based costing, retention in antiretroviral therapy care, Malawi, patient tracing, prevention of treatment interruption

## Abstract

**Introduction:**

Antiretroviral therapy (ART) improves the health of people living with HIV (PLHIV). However, a high loss to follow-up, particularly in the first year after ART initiation, is problematic. The financial expenses related to client retention in low- and middle-income countries (LMICs) in sub-Saharan Africa are not well understood. This study aimed to comprehensively assess and quantify the financial costs associated with routine ART retention services for clients in their first 12 months in care at Lighthouse Trust’s (LT) Martin Preuss Centre (MPC), a large, public ART clinic in Lilongwe, Malawi.

**Methods:**

We performed activity-based microcosting using routine data to assess the expenses related to routine ART retention services at the MPC for 12 months, from January to December 2021. MPC provides an “ART Buddy” from ART initiation to 12 months. The MPC’s Back-to-Care (B2C) program traces clients who miss ART visits by at least 14 days. Clients may be traced and return to care multiple times per year. We assessed client retention costs for the first 12 months of treatment with ART and conducted a sensitivity analysis.

**Results:**

The total annual cost of ART retention interventions at MPC was $237,564. The proactive Buddy phase incurred $108,504; personnel costs contributed $97,764. In the reactive B2C phase, the total cost was $129,060, with personnel expenses remaining substantial at $73,778. The Buddy unit cost was $34 per client. The reactive B2C intervention was $17 per tracing event. On average, the unit cost for ART retention in the first year of ART averaged $22 per client.

**Conclusion:**

This study sheds light on the financial dimensions of ART retention during the first year on ART among clients in a routine public setting. ART retention is both costly and critical for helping clients adhere to visits and remain in care. Continued investment in the human resources needed for both proactive and reactive retention efforts is critical to engaging and retaining patients on lifetime ART.

## Introduction

Antiretroviral therapy (ART) has demonstrated remarkable efficacy in mitigating HIV/AIDS-related morbidity and mortality, particularly in resource-limited settings [1, 2]. However, the full realization of its benefits has been impeded by the high loss to follow-up (LTFU) [3], especially within the first year of ART [4]. Newly initiated ART clients are particularly vulnerable to treatment interruptions due to a multitude of factors, including the severity of their illness, difficulties in disclosing their HIV status, and adaptation to life with HIV and ART [5, 6]. Estimates across sub-Saharan Africa (SSA) report an average of 65% retained in care at 36 months [3, 7]. Despite the critical importance of client retention, only a few studies have explored the costs of retaining clients in routine ART care in low- and middle-income countries (LMICs) in SSA [8–13].

The Lighthouse Trust (LT), a national ART Center of Excellence in Lilongwe, Malawi, operates with the Malawi Ministry of Health (MoH) to provide HIV care, treatment, and support across Malawi [14]. In its two urban flagship clinics in Lilongwe, the LT serves more than 35,000 clients on ART: 24,000 at the Martin Preuss Centre (MPC) and 11,000 at Lighthouse (LH) [15]. Clients at all LT clinics receive the same services, including integrated care, retention support, and clinical management, using an electronic medical records system (EMRS) [16]. Since 2006, LH and MPC have implemented a client retention program, “Back-to-Care” (B2C), that traces ART clients who miss a clinic visit by ≥ 14 days by phone or a home visit. B2C plays a critical role in reaching and retaining LT clients in care [17–19]. B2C is also reactive, waiting for clients to miss visits before intervention. In 2016, in response to growing concern about treatment interruption during the early stages of treatment, LT also introduced the Start Safely to ART (START) program in 2020. This initiative pairs all newly initiating ART clients with Expert Client *Treatment Buddies. Buddies* are HIV-positive peer mentors who provide vital psychosocial support and closely monitor up to 15 clients during the first 12 months of critical ART initiation.

The primary goal of this costing study is to conduct a comprehensive assessment and quantification of the financial cost associated with routine ART retention services among new ART initiates at MPC during 2021. Understanding the financial implications of proactive and reactive ART retention interventions at a large, public ART clinic in Lilongwe, Malawi will contribute to the broader discourse regarding both retention and ART program sustainability. The findings may also help identify potential areas for cost optimization and improvements in resource allocation at MPC and other public ART clinics in LMIC settings.

## Methods

### Objective

This comprehensive cost study aimed to improve the understanding of the financial and economic implications of routine proactive and reactive retention interventions for clients in their first 12 months on ART at the MPC clinic in Lilongwe, Malawi.

### Setting: Lighthouse Trust’s (LT) Martin Preuss Centre (MPC)

MPC is the largest public provider of ART services in Malawi. LT umbrella policy and practice are the same across clinics, including all retention interventions. LT staff rotate between the MPC and LH locations as needed. All client data is managed in real-time using the EMRS. ART clinic visits for new ART initiates are scheduled monthly during the first six months and then every three or six months if the patient is stable and adherent. B2C forms, including location information via phone and address details for tracing, are collected at initiation and ideally updated annually. As an indication of MPC patient volume, between April and June 2023, of 18,842 scheduled ART visits, 1798 (~ 10%) missed visits by ≥ 14 days and were referred to B2C.

### Client retention programs

#### Proactive efforts before a visit or within 13 days of missed visits: The ART Buddy program

ART patients receive more intense, proactive retention support from ART initiation through 12 months, alongside routine B2C. Following the initiation process, newly enrolled clients receive support from Expert Client “Buddies” during their initial 12 months of care. Expert clients have ~ 15 new ARTs available for support. All new ART clients are expected to have an initial encounter with their assigned Buddy on ART initiation day and continue to receive support from their Buddy through 12 months on ART only. These buddies remind clients of their scheduled ART visits via phone calls and follow up with clients immediately after a missed visit, within 1–13 days of the appointment. Buddies also update locator forms for clients who change contact information, such as phone numbers or home locations. If clients fail to report for any scheduled visit by 14 or more days, they are referred to B2C. Expert client budget services are only provided for clients during their first year on ART.

#### Reactive retention efforts after a missed visit ≥ 14 days: Back-to-care (B2C)

B2C traces all clients who missed visits by ≥ 14 days in accordance with MoH policy, including new initiates. [20] EMRS is used to identify and refer potential LTFU clients to tracing. A dedicated team of B2C tracers manually reviewed the LTFU list to identify and correct any errors in the EMRS data, removing people who attended visits from the tracing list. Clients with completed locator forms are initially traced by phone through SMS or calls, with up to five attempts made, and if necessary, up to three home visits are attempted. In cases where clients are successfully reached, the B2C team of field tracers and/or health promoters encourages those who have missed appointments or defaulted on treatment to return to care. The B2C team also conducts semiformal interviews with clients to assess the outcomes of their ART treatment and records this information on paper-based B2C forms, which data clerks subsequently input into the EMRS. The EMRS helps determine if and when a client returns to care, allowing for the cessation of B2C client follow-up for that specific event or month. Each client may have a B2C event for any visit; clients may have multiple B2C events per year.

### Data collection

In adherence to the Global Health Cost Consortium Reference Case guidelines [21], we performed activity-based microcosting to assess the expenses related to routine ART retention activities at the MPC clinic in Lilongwe, Malawi. Our data encompassed both financial and economic cost estimations for all resources and activities essential for executing the routine retention intervention. Cost information was obtained from the MPC expenditure records, payroll information, and procurement records. We used routine program data to estimate the number of ART clients retained and the number of tracing events in 2021. The financial costs accounted for the direct expenses incurred in the process of retaining ART clients, whereas the economic costs took into consideration the opportunity cost linked to overhead expenses. Since the perspective of the analysis was from the LT organizational perspective (payer), we excluded costs that were not incurred by the clinic, such as medication costs, which are paid by the government, and study-specific personnel that would not be transferable to routine program implementation.

### Data analysis

We categorized our cost data into two main groups: fixed costs and recurrent costs (as shown in Table 1). Fixed costs encompassed specific activities such as the initial training of retention personnel, a one-time motorcycle insurance premium payment, text messaging system subscriptions, and the procurement of equipment and motorcycles. These fixed expenses were incurred only at the outset of the intervention when the equipment was expected to have a useful life of 5 years. In contrast, variable costs were essential for sustaining the intervention over time. These variable expenses were further subdivided into distinct input categories, including personnel costs, communication expenses for reaching and following up with ART clients, general office supplies, motorcycle maintenance for client tracing, fuel costs, protective gear for motorcycle riders, and overhead costs representing opportunity costs.

For equipment costs in 2021, we applied a discount rate of 3% over an assumed lifespan of 5 years. To calculate the unit cost of the proactive Buddy intervention, we divided the total expenses incurred during a two-week period by the number of new ARTs initiated in 2021. Conversely, the unit cost for the reactive B2C intervention was determined by dividing the total expenses incurred for ART retention beyond the initial two weeks by the number of tracing events.

All costs were converted from Malawi Kwacha (MWK) to US dollars using the 2021 exchange rate of 1$=825 MWK. Our analyses were conducted using Microsoft Excel (version 16.76; Microsoft, Redmond, WA). We also conducted a sensitivity analysis to assess how changes in personnel costs, a significant component of the intervention’s expenses, might impact overall costs. This analysis was prompted by the inherent challenge of distinguishing personnel expenses related to ART intervention from those associated with routine care.

## Results

### Visits and tracing events among new ART initiates at MPC

In 2021, there were 3,280 new ART clients initiated at MPC. Among these clients on ART, 7,588 had tracing events.

### Retention costs

The total cost of ART retention interventions at the MPC is $237,564 (Table 2). The early retention buddy phase incurred a total cost of $108,504, with personnel costs being the most significant at $97,764, followed by training at $6,592. In the reactive retention B2C phase, the total cost was $129,060, with personnel expenses remaining substantial at $73,778. Overhead, fuel and vehicle costs emerged as significant contributors, amounting to approximately $12,518, $10,427, and 9,105, respectively.

### Retention cost categories

Table 3 provides a comprehensive breakdown of the fixed and variable costs associated with ART retention during the first year in care at MPC, classified into proactive Buddy and reactive B2C interventions. For the Buddy activities, the fixed (start-up) costs amounted to $9,647, representing 9% of the total cost, while the variable (recurrent) costs were significantly greater at $98,587, accounting for 91% of the total cost. The overall cost of early intervention was $108,504. In contrast, B2C incurred higher fixed (start-up) costs at $16,757, comprising 13% of the total cost, with variable (recurrent) costs of $112,303, making up 87% of the total cost. The total cost of B2C was $129,060. This breakdown offers valuable insights into the allocation of resources and the financial aspects of ART retention care at the MPC.

### Per client unit cost

The unit cost of ART retention care at the MPC for the Buddies, covering care for 3,280 new clients, was $34 (Table 4). In contrast, B2C, with 7,588 tracing events, yielded a lower unit cost of $17. Combining both Buddies and B2C, the overall unit cost for ART retention care at the MPC in 2021 averaged $22 per client/tracing event.

### Cost drivers

**Figure 1:**
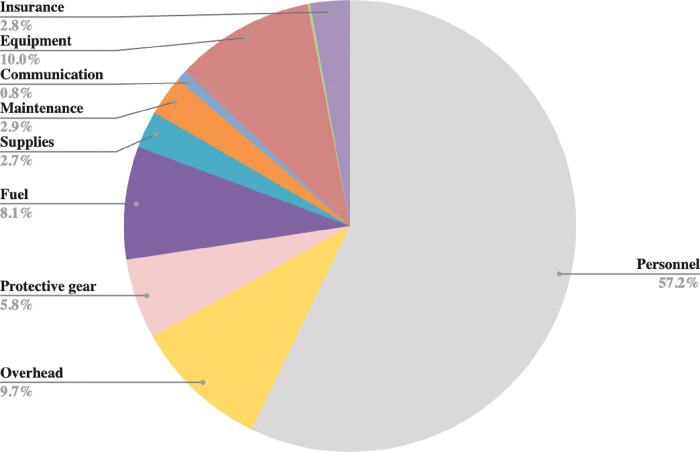
Cost drivers for proactive ART retention intervention: Buddies

**Figure 2:**
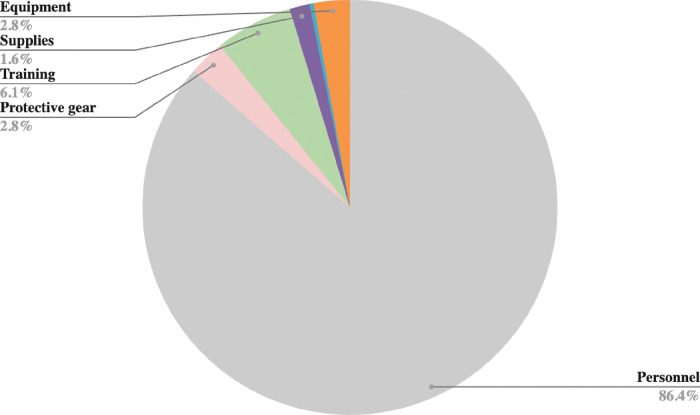
Cost drivers for reactive ART retention intervention: B2C

Figures 1–2 provide an overview of the primary cost drivers for ART retention intervention in both the Buddy and B2C phases. In the proactive Buddy intervention (Fig. 1), personnel costs constitute the largest portion, accounting for 86% of the total expenses, while training and protective gear costs represent 6% and 3%, respectively. Moreover, during the reactive B2C intervention, personnel costs remained substantial but decreased to 57% (Fig. 2). Overhead and equipment costs become more prominent at 10% each, followed by fuel and protective gear costs, which make up 8% and 6%, respectively.

### Sensitivity analysis

Considering the significant impact of personnel costs on both proactive Buddy and reactive B2C retention interventions, coupled with the ongoing trend of rising personnel expenses, the need for sensitivity analysis becomes paramount. We performed a univariate sensitivity analysis to evaluate the total and unit costs of the interventions. When adjusting for a 25% increase in personnel costs, the proactive Buddy intervention increased from $108,000 to $136,000, a unit cost increase in Buddies from $33 to $42. Similarly, the cost of B2C intervention also increased from $129,000 to $147,000, resulting in a per-tracing cost increase from $17 to $19. The findings indicate that the proactive Buddy intervention is more personnel-intensive and susceptible to changes in personnel costs than the B2C intervention, as evidenced by the greater increase in cost per client retained.

## Discussion

In this study, we provide a comprehensive breakdown of the routine costs associated with proactive and reactive ART retention interventions for clients in their first year on ART at the large, public ART clinic in Lilongwe, Malawi. This cost analysis provides valuable insights into the financial aspects of an ART retention intervention conducted in a resource-constrained setting. In the proactive Buddy program, expenses totaled $108,504, with personnel costs being the largest contributor. The late retention program, B2C, incurred a total cost of $129,060, where personnel expenses remained substantial but overhead, fuel and vehicle expenses also played a significant role. The study highlights the critical cost drivers across retention phases, offering important information for LMIC policymakers and healthcare administrators to consider for retention service allocations and program planning.

Although this analysis was not specifically a cost-effectiveness analysis, the unit cost analysis offers insights into the drivers of effective retention interventions. For the proactive Buddy retention program, the unit cost per client (covering 3,280 new clients) was $34, while the late retention program had a lower unit cost of $17 per tracing event (involving 7,588 tracing episodes). Although these numbers might suggest that proactive Buddy retention is more expensive and therefore less cost-efficient, this may be misleading. Early retention initiatives such as Buddies prevent or reduce the likelihood of missed visits, keep contact information updated, and may help foster engagement in care beyond the first 12 months when Buddy supports sunset. Moreover, these findings suggest that the $17 per tracing event is likely a reasonable cost to return and retain clients in care, suggesting continued investment in B2C. Overall, it appears that the combination of proactive and late program retention activities may be the most cost-efficient model, averaging $22 per client/tracing event. The average retention cost, $22, may serve as a valuable benchmark for evaluating cost efficiency and informing resource allocation decisions.

Retention efforts are recognized as critical but costly aspects of quality ART programs at scale. However, retention efforts at the MPC are lower, or far lower, than those reported previously, suggesting cost efficiency. For example, a recent costing study of three HIV retention models in SSA found that improving ART retention by 25% could cost between $93 and $6518/client [8]. These retention costs pose sustainability challenges. MPC costs are more in line with lower-cost retention models, with retention efforts at $36.56 USD per client. According to a community-based tracing model in Tanzania, client tracing services had a unit cost of $47.56 USD, while support for the client returning to care was $206.77 USD. For tracing services, B2C has a lower cost than this lower-impact model [22].

Retention intervention costs at the MPC should not be used to overlook pervasive and persistent funding gaps that reduce Buddy or B2C effectiveness. Although retention at LT clinics, including the MPC, is consistently more than 75% at 12 months, retention at LT clinics still falls short: an average of 63% of ART clients are retained at 24 months. This is far below the 90% retention target needed for client VLSs and epidemic control. [22]Second, current resources provide resources only for clients during their first 12 months of care, shortchanging clients who may benefit from longer-term support. Third, additional resources are needed to help update accurate location information. In 2021, 1,803 clients (29%) remained untraceable due to a lack of actualized address information, preventing efforts to return clients to care. Furthermore, approximately 1,184 clients (19%) returned to the facility after the tracing list was generated and verified, leading to wasted tracing resources. Finally, gaps in B2C grow as client volume increases while funds decrease [23]. At the MPC clinic from April to June 2023, only 40% (719/1798) of potential LTFU patients were successfully identified. Additional proactive retention efforts, such as LT’s recent two-way texting system to improve early retention support [24, 25], are needed to reduce LTFU before it happens.

### Limitations

Lighthouse Trust is a Centre of Excellence with highly motivated and capable staff; therefore, not all costs would be the same in other LMIC ART clinics. Lighthouse also has complementary peer support groups in the community that enrich the client experience; these costs were outside the scope of retention-specific activities but are likely to improve engagement in care. Other LT-specific activities, like call center services designed to enhance client support and engagement, psychosocial counseling, and referral services for clients who have been exposed to gender-based violence are not retention-specific but help clients engage in care. These costs were not included, potentially leading to retention underestimates. Moreover, the analytic approach has several limitations due to the use of routine data and funding limitations, including the focus on a single-center analysis, the assumption of linear sensitivity, the reliance on clinical records, and the absence of time-in-motion analysis to estimate the actual personnel cost. Finally, some clients may miss more visits, costing more to retain. In this financial costing, we explored overall costs for the first 12 months, but should explore the costs of retaining different client types, by retention patterns, in the future. Despite these limitations, this study provides valuable insights into the financial aspects of ART retention interventions during clients ‘first year on ART at MPC, emphasizing the critical role of personnel expenses and the distribution of fixed and variable costs across both the proactive and late program stages.

## Conclusion

These findings significantly contribute to our understanding of the financial landscape surrounding ART retention interventions, especially during the first year on ART. To ensure the long-term sustainability and efficiency of the ART retention care program at the MPC, it is imperative to explore resource optimization strategies for both proactive and late retention programs while maintaining continuous cost monitoring and evaluation. To improve retention efficiency, focusing scarce retention resources on clients at the highest risk of LTFU and during time periods when the risk of LTFU is highest (e.g., ART initiation) may be advisable. Additionally, linkages between EMRSs between facilities, such as via a National Health Management Information System (HMIS), could reduce the impact of silent transfers (clients moving clinics informally) and those who receive emergency ART supplies while traveling. Overall, these results reinforce calls for healthcare policymakers and administrators to continue to advocate for retention resources to ensure the wellness of both PLHIV on ART and the overall ART program.

## Figures and Tables

**Table 1:** Cost Categories

Inputs	Cost category	Description
Personnel	Variable	Covers personnel salaries, benefits, and the time and effort invested in retention activities.
Communication	Variable	Expenses related to phone services from various companies.
Protective gear	Variable	Gear for motorcycles, such as helmets and other safety equipment.
Fuel	Variable	Fuel costs associated with the motorcycles used for B2C retention efforts.
Maintenance	Variable	Expenses incurred in maintaining the motorcycles.
General supplies	Variable	Supplies used for documentation and communication with clients.
Overhead	Variable	The costs related to utilities and building maintenance.
Equipment	Fixed	Investments that have a lifespan exceeding one year, including mobile phones, desktops, furniture, and motorcycles.
Training	Fixed	Startup expenses allocated for training staff in client retention strategies.

**Table 2:** Activity and input cost of ART retention care at the MPC in 2021 (USD)

Intervention	Cost category	Total cost	% of total cost
Proactive: ART Buddy	Fixed costs		
	Training	$6,592	5%
	Equipment	$3,055	2%
	Variable costs		
	Personnel	$93,764	86%
	Protective gear	$3,055	3%
	Supplies	$1,689	1%
	Communication	$349	1%
Total Buddy intervention costs		108,504	100%
Reactive: B2C	Fixed costs		
	Equipment	$12,853	10%
	Training	$3,636	3%
	Insurance	$268	1%
	Variable costs		
	Personnel	$73,778	57%
	Overhead	$12,518	10%
	Fuel	$10,427	8%
	Protective gear	$7,422	5%
	Maintenance	$3,685	3%
	Supplies	$3,459	2%
	Communication	$1,018	1%
Total B2C intervention costs		$129,060	100%
Overall, ART retention costs		$237,564	

**Table 3:** Fixed vs variable cost of ART retention care at the MPC in 2021 (USD)

Intervention	Cost category	Total cost	%of total
Proactive: ART Buddy	Fixed (Startup)	$9,647	9%
	Variable (Recurrent)	$98,587	91%
	Total	$108,504	100%
Reactive: B2C	Fixed (startup)	$16,757	13%
	Variable (Recurrent)	112,303	87%
	Total	129,060	100%
Total		$237,564	

**Table 4:** Unit cost of ART retention care at the MPC in 2021 (USD)

Intervention	Total cost	Number of clients/Tracing event/	Unit cost
Proactive Buddy	$108,504	3,280*	$34
Reactive B2C	$129,060	7,588**	$17
Total	$273,298	10, 868	$22

* Number of new ART clients in 2021,

** Number of tracing events

## Data Availability

The full data set used for this costing study is included as supplementary material.
